# Diabetes mellitus *HNF4A*-MODY in children from the Russian population: clinical and genetic features

**DOI:** 10.3389/fendo.2025.1673182

**Published:** 2025-10-01

**Authors:** Elena A. Sechko, Dmitry N. Laptev, Mariia P. Koltakova, Rita I. Khusainova, Ildar R. Minniakhmetov, Tamara L. Kuraeva, Irina A. Eremina, Elena V. Titovich, Olga B. Bezlepkina, Valentina A. Peterkova

**Affiliations:** Endocrinology Research Centre, Moscow, Russia

**Keywords:** gene HNF4A, monogenic diabetes mellitus in children, MODY, NGS, MODY1

## Abstract

**Introduction:**

*HNF4A*-MODY is a rare subtype of MODY in children that requires treatment. The clinical features of children with *HNF4A*-MODY are limited. Adult patients with *HNF4A*-MODY are treated with insulin, diet, oral antidiabetic drugs, and incretin drugs. Our cross-sectional study presents the clinical features of 15 probands with genetically confirmed *HNF4A*-MODY from the pediatric registry of MODY in Russia.

**Materials and methods:**

This study presents the genetic, clinical, and laboratory characteristics of 15 children with *HNF4A*-MODY in the Russian population.

**Results:**

The frequency of *HNF4A*-MODY was 1.8%, 95% CI [1.0, 3.0] among all pediatric MODY cases (*n* = 15/807) in Russia. The median age at diagnosis was 12.8 years [12.1, 14.0]. Hyperglycemia was diagnosed incidentally in 71.5% of cases. Glycated hemoglobin (HbA1c) was 8.0% [7.0, 9.2]. At birth, macrosomia was present in 35.7% of patients and hypoglycemia in 7%. Family history was positive in 57.1%, with DM diagnosed in first-degree relatives at age 29 [27.3, 32.8] years in 50% of cases, which was significantly different from the age of DM diagnosis in their children (*p* < 0.05).

**Discussion:**

We examined patients with *HNF4A*-MODY with a duration of 1.2 [0.8, 1.9] years. The degree of hyperglycemia in all patients met the diagnostic criteria for DM. Molecular genetic testing revealed a high percentage of deletions and nonsense variants (28.5% each). 64.5% of patients were prescribed drug therapy (21% insulin, 43% metformin) at the onset of diabetes. Forty-three percent of patients were transferred successfully to sulfonylurea therapy (including patients with complete insulin withdrawal) following genetic testing and *HNF4A*-MODY verification. The attempt to switch from insulin to sulfonylurea drugs was unsuccessful due to significant glycemic deterioration in one case.

## 1 Introduction

The term **“**maturity-onset diabetes of the young**”** (MODY) was first introduced by Tattersall and Fajans in 1974–1975 (1, 2). Tattersall, R.B. (1974) described a prospective observation of the RW family beginning in 1958 in his publication. This family included 360 members in 6 generations, with DM diagnosed in 74 individuals. He noted the autosomal dominant mode of inheritance of insulin-independent diabetes and characteristic features, including mild hyperglycemia, an early onset of DM, usually before age 25 years, and a successful treatment with sulfonylurea (SU) medications (1). Then, Fajans S.S. and Brown M.B. demonstrated an effective treatment with SU medications in the descendants of this family in 1993 (3).

In 1996, Kazuya Yamagata et al. (4) revealed a genetic cause of diabetes in the descendants of this family—nucleotide sequence variants in the *HNF4A* gene. Later on, a decreased glucose-stimulated insulin secretion was demonstrated in such patents (5). This subtype received the name of MODY1 or *HNF4A*-MODY (4).

Hepatic cell nuclear transcription factor 4α (HNF4α) participates in embryogenesis and development of hepatic cells (6) and the intestinal tract (7). It also regulates the metabolism of cholesterol and fatty acids (8), leading to the progression of concomitant conditions in some patients with *HNF4A*-MODY.

The *HNF4A*-MODY frequency varies in different populations in different countries and it is the third most common subtype of MODY (9) after *GCK-* and *HNF1A-*MODY (10) and accounts for about 10% in the general population, including children and adults (9). But it is a rare subtype of MODY (11) in the pediatric patient cohort, so clinical feature data of *HNF4A*-MODY in children are limited. In a study of young people under 20 years, *HNF4A*-MODY was diagnosed in 7 of 47 participants (12), which amounted to about 15%. However, those researchers conducted genetic testing for only three of the 14 subtypes of MODY. This article presents clinical and laboratory features and results of genetic testing in 15 probands with *HNF4A*-MODY under 18 years of age from Russia.

## 2 Materials and methods

### 2.1 Data collection

Pediatric patients diagnosed with MODY are observed at the Endocrinology Research Center from all over Russia. By January 2025, the Russian registry of children with MODY diagnosis included 807 probands (index cases) in the age under 18 years.

Based on the genetic research results, 15 nucleotide sequence variants of the *HNF4A* gene were identified. However, the variant of c.439G>A (p.Val147Ile) was classified as benign, and this patient was excluded from the calculation of the study results. Thus, the study results contain the analyzed data from 14 probands with *HNF4A*-MODY confirmed by genetic testing. The examination of the patients was conducted within the period between January 2021 and January 2025.

Genetic testing was performed in patients who had an established DM diagnosis at the age of from 6 months to 18 years with negative multiple insular autoimmune antibodies (AAB) and one or more of the following features:

1. Diabetes in the family medical history;
2. Secretion of C-peptide >0.5 ng/ml
3. Insulin dose <0.5 U/kg/day

#### 2.1.1 Study design

Single-center observational, diametrical, single-sample, single-arm study.

### 2.2 Methods

The genomic DNA (gDNA) was extracted from peripheral blood lymphocytes using the MagPure Blood DNA kit (Magen, Guangzhou, China). Quantity and purity of the extracted gDNA were assessed using a Nanodrop 2000 spectrophotometer (Thermo Fisher Scientific, Waltham, MA, USA) and a Qubit 2.0 fluorometer (Invitrogen, Carlsbad, CA, USA) with the Qubit dsDNA HS Assay Kit.

Sequencing was performed on the Illumina platform using the paired terminal sequencing method (2 × 150 bp). Average coverage depth was 173.4×, with 99.88% of targeted nucleotides achieving an effective coverage of >10×.

Preparation of the whole-genome library (KAPA HyperPlus, Roche, Switzerland) and enrichment of the targeted DNA (KAPA HyperCapture, Roche, Switzerland) were performed in accordance with the manufacturer’s protocols. The custom panel was aimed at 27 genes coding regions: *GCG, GLUD1, WFS1, HNF1A, GCK, INS, HNF1B, ABCC8, HNF4A, RFX6, PTF1A, NEUROD1, AKT2, ZFP57, INSR, EIF2AK3, PPARG, PAX4, PDX1, GLIS3, KCNJ11, SLC16A1, FOXP3, BLK, CEL, KLF11, SCHAD*, *GCGR*, which have been extensively described in the scientific literature and documented in the OMIM database as linked with monogenic forms of DM.

The study utilized a next-generation sequencing (NGS) on the Illumina Novaseq 6000 platform (Illumina, San Diego, CA, USA) with paired reads (2 × 100 bp). Processing of the NGS data was performed with the use of a standardized automated conveyor, including the reads alignment with the human genome reference sequence (GRCh38), post-alignment, variants identification, and quality filtration. The detected variants were annotated for all known gene transcripts using the RefSeq database and disease-evoking power prediction algorithms according to recommendations of the American College of Medical Genetics and Genomics. Chromosomal microarray (CMA) was performed with the use of the chips (CytoScan HD Accel Array) on the Genoscan 300 device (Russia).

Clinical characteristics included medical history, family medical history, and anthropometric parameters. The BMI (kg/m²) was calculated as ratio of the body weight (kg) to the squared height (m²), assessed according to the WHO standards for specific age and sex, and presented as a standard deviation from the mean value (SBS stands for the standard bias score). The obesity diagnostic criterion was SDS of the BMI > 2.0 (WHO, 2007). The macrosomia diagnostic criterion was the birth weight of >4000 g with birth in time or >90th percentile according to fetal growth tables for the given gestational age (13) (the INTERGROWTH-21 score was used).

The insular autoimmune antibodies were detected, including GADA, IAA, ICA, IA-2A, and ZnT8A.

Laboratory parameters of the patients were examined, including levels of the glycated hemoglobin (HbA1c), glucose, C-peptide, and insulin.

Evaluation of the hyperglycemia degree, insulin, and C-peptide secretion was performed during the oral glucose tolerance tests (OGTT). If the level of HbA1c exceeded 7% and/or there was an increase in glycemia during the day of more than 11.1 mmol/l, stimulated secretion of C-peptide and insulin was studied when performing a test with a standard liquid breakfast (based on the calculation of 0.7 g of carbohydrates/kg).

Presence of the insulin resistance (IR) was assessed using the HOMA-IR (homeostasis model assessment) index with the use of a standard formula: (IRI0×Gl0)/22.5, where IRI is immunoreactive insulin, μU/ml; Gl is glucose, mmol/L. IR was recorded when the HOMA index value was >3.2.

### 2.3 Statistical analysis

Statistical processing of the obtained data was performed with the use of the statistical software package Statistica 8.0 (StatSoft, USA) and MS Excel 2010 (Microsoft, USA). The data are presented as the median value and interquartile range (Me [25; 75 percentile]).

In order to compare two independent samples by their quantitative characteristics, the Mann–Whitney test was used, and for qualitative characteristics, the chi-square test (χ2) was used. The critical level of significance of differences was accepted at *p* < 0.05.

### 2.4 Institutional review board statement

This study was conducted in accordance with the principles of the Declaration of Helsinki and approved by the Ethics Committee of the Endocrinology Research Centre (protocol code No. 16 dated 13 September 2023). The study protocol was approved by the local Ethics Committee of the Federal State Budgetary Institution of the “National Medical Research Center of Endocrinology” of the Ministry of Health of the Russian Federation (protocol No. 169 dated 13/09/2023).

## 3 Results

The Russian registry of children with MODY includes 807 children (index cases), including 15 probands from different families with *HNF4A*-MODY. Thus, the frequency of *HNF4A*-MODY in the pediatric cohort of patients with MODY was 1.8%, with a 95% confidence interval of [1.0; 3.0]. This study presents data from the patients with *HNF4A*-MODY only (**Table 1**).

**Table 1:** Clinical and laboratory features of patients with *HNF4A*-MODY.

Feature	Value	Reference values
Age of the genetic test, years	14.5 [13.8, 15.4]	–
Duration of hyperglycemia, years	1.2 [0.8, 1.9]	–
SDS BMI	0.9 [−0.3, 2.1]	-1SD - +1SD
HbA1c, %	8.1 [7.1, 8.8]	4–6
Total cholesterol, mmol/l	4.3 [3.9, 4.5]	3.3–5.2
HDL, mmol/l	1.1 [0.9, 1.2]	0.90–2.60
LDL, mmol/l	2.7 [2.4, 2.9]	1.10–3.00
TG, mmol/l	0.8 [0.7, 1.2]	0.1–1.7
Results of mixed-meal test in patients with *HNF4A*-MODY		
Blood Glucose, mmol/l, 0 min	6.4 [5.0, 7.7]	3.3–5.5
Blood Glucose, mmol/l, 60 min	13.7 [10.1, 16.2]	–
Blood Glucose, mmol/l, 120 min	13.2 [11.2, 16.8]	<7.8 – norm, 7.8–11.0 – IGT, >11 – DM
Insulin, µmol/l, 0 min	7.4 [5.25, 12.1]	2.0–24.9
Insulin, µmol/l, 60 min	36.1 [30.0, 47.2]	–
Insulin, µmol/l, 120 min	31.2 [22.0, 37.3]	–
C-Peptide, ng/ml, 0 min	1.9 [1.3, 2.5]	1.1–4.4
C-Peptide, ng/ml, 60 min	5.3 [5.1, 5.8]	–
C-Peptide, ng/ml, 120 min	5.0 [4.4, 5.2]	–

SDS BMI, body mass index standard deviation score; HDL, high-density lipoproteins; LDL, low-density lipoprotein; TG, triglycerides, IGT, impaired glucose tolerance; DM, diabetes millitus.

### 3.1 Onset of *HNF4A*-MODY

The median age when detecting hyperglycemia and diagnosing DM was 12.8 years [12.1; 14.0]. Hyperglycemia was diagnosed incidentally in 71.5% (*n* = 10) during preventive examinations, including 7% (*n* = 1) of patients examined due to an aggravated family medical history of DM. Clinical symptoms of DM were observed in 28.5% (*n* = 4), including ketosis in one patient. At the time of diagnosis, the level of glycemia was 8.4 mmol/L [7.5; 13.3], and HbA1c was 8.0% [7.0; 9.2].

### 3.2 Neonatal history of the patients with *HNF4A*-MODY

The medians of gestational age were 40 weeks [38.3; 40], height—53 cm [50; 54] (SDS 1.6 [1.0; 2.5]), and body weight—4915 g [3282.5; 4712] (SDS 1.9 [0.9; 2.9]). Fetal macrosomia at birth was observed in 35.7% (*n* = 5) of patients. One patient (7%) had hyperinsulinemic hypoglycemia in the neonatal period.

### 3.3 Family history of patients with *HNF4A*-MODY

DM was diagnosed in 57.1% (*n* = 10) of the proband’s family members, including the cases with DM in three generations in 35.7% (*n* = 5). In 50% (*n* = 7) of cases, DM was diagnosed in first-degree relatives. In 35.7% (*n* = 5) of parents the *HNF4A*-MODY was confirmed by genetic testing, and two additional parents had a clinical diagnosis of type 2 DM (DM2). The age at the time of DM diagnosis in parents was 29 [27.3; 32.8] years, which was significantly different from their children (*p* < 0.05). All parents with DM received different anti-hyperglycemic medications: two SU medications, two metformins, one sodium-glucose cotransporter 2 inhibitor (SGLT-2i), and two insulins. Combined therapy (SGLT-2i, metformin, and SU medications) was required in a case.

### 3.4 Current examination data of the patients with *HNF4A*-MODY

The age of patients at the moment of examination (when the genetic testing was performed) was 14.5 years [13.8; 15.4], the disease duration was 1.2 years [0.8; 1.9], and the SDS of the BMI was: +0.9 [−0.3; 2,1]. Among patients, 14% were overweight and 29% had obesity. The level of HbA1c was 8.1% [7.1; 8.8] (**Table 1**).

Glycemia values met the diagnostic criteria of DM in all cases. The insulin and C-peptide secretion was maintained in all patients. Results of the stimulated insulin and C-peptide secretion are given in **Table 1**. The IR, according to the HOMA-IR value, was diagnosed in 29% (*n* = 4) of cases.

The levels of total cholesterol, triglycerides, and high-density lipoproteins (HDL) were within reference values in all patients. The levels of low-density lipoproteins (LDL) were elevated in two patients (14.3%). The insular autoimmune antibodies (GADA, ICA, IAA, IA-2A, and ZnT8A) were negative in all patients.

### 3.5 Results of genetic testing of the patients with HNF4A-MODY

**Figure 1:**
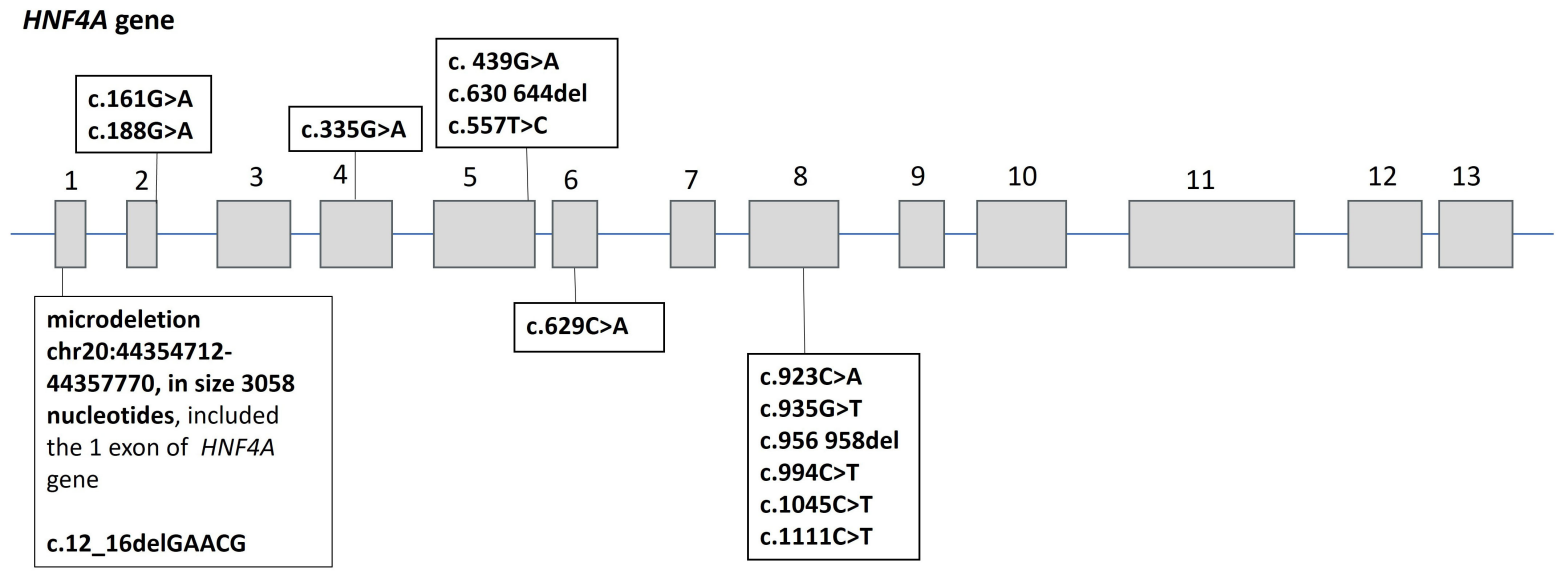
Spectrum of nucleotide sequence variants in the *HNF4A* gene in 15 probands with *HNF4A*-MODY. Numbers 1–13 indicate exons of the *HNF4A* gene.

Fifteen heterozygous variants of the nucleotide sequences of the *HNF4A* gene were identified in 15 families (**Figure 1**):46.6% missense sequences (*n* = 7), 26.7% nonsense sequences (*n* = 4), and 26.7% deletions (*n* = 4). 85.7% (*n* = 12) were pathogenic or likely pathogenic (**Tables 2**, **3**) out of all nucleotide sequence variants. The variant of p.Thr211_Met215del was categorized as a variant of unknown clinical significance (VUS). However, this variant was found in two brothers and sisters and their mother with the MODY phenotype (negative insular autoimmune antibodies, preserved C-peptide secretion, aggravated family medical history of DM, and the same variant confirmed in their parent, having mild diabetes manifestations, as well as the effectiveness of treatment with SM derivatives), which permitted us to classify the variant of p.Thr211_Met215del as a MODY-causing one. A variant of c.439G>A (p.Val147Ile) was categorized as benign; this patient was excluded from the analysis of clinical and laboratory results of the study.

**Table 2:** Genetic features of patients with single-nucleotide substitutions (SNP) in the *HNF4A* gene.

№	*n*	cDNA, variant	Protein, variant	ACMG classification	Allele frequency (gnomAD v4.1.0)	CADD phred	In-silico individual predictions (varsome)*		
							Pathogenic	Uncertain	Benign
1	1	c.161G>A:p.S54N	p.Ser54Asn	Likely pathogenic	–	27.50	32	5	1
2	1	c.188G>A	p.Arg63Gln	Pathogenic	–	32.00	29	5	0
3	1	c.335G>A	p.Arg112Gln	Pathogenic	3.719e-06	32.00	25	6	0
4	1	c. 439G>A	p.Val147Ile	Benign	9.59e-04	10.85	2	3	32
5	1	c.557T>C	p.Phe186Ser	Pathogenic	–	27.90	32	5	0
6	1	c.629C>A	p.Ala210Asp	Likely pathogenic	–	25.20	12	10	0
7	1	c.994C>T	p.Gln332*	Pathogenic	–	37.00	4	4	1
8	1	c.935G>T	p.Arg290Leu	Pathogenic	–	33.00	22	6	–
9	1	c.1045C>T	p.Gln349*	Pathogenic	–	41.00	8	4	1
10	1	c.1045C>T	p.Gln349*	Pathogenic	–	41.00	8	4	1
11	1	c.923C>A	p.Ser308*	Pathogenic	–	36.00	2	3	3

cDNA, complementary DNA; ACMG Classification, American College of Medical Genetic Classification.

For CADD, we classified variants based on the phred-like score with a cutoff of 20, below which variants were classified as benign and otherwise deleterious, as suggested by the authors.

*AlphaMissense, BLOSUM, BayesDel, addAF, BayesDel, noAF, DANN, DEOGEN2, EIGEN, EIGEN-PC, EVE, FATHMM, FATHMM-MKL, FATHMM-XF, LIST-S2, LRT, M-CAP, MVP, MaxEntScan, MetaLR, MetaRNN, MetaSVM,MitImpact, MitoTip, MutPred, MutationAssessor, MutationTaster, PROVEAN, Polyphen2-HDIV, Polyphen2-HVAR, PrimateAI, REVEL, SIFT, SIFT4G, phastCons100way, vertebrate, phyloP, scSNV-ADA, scSNV-RF (https://varsome.com/about/resources/germline-implementation/#insilicopredictions)

**Table 3:** Genetic characteristics of patients with deletions in the *HNF4A* gene.

№	*n*	cDNA, variant	Protein, variant	ACMG classification
1	1	chr20-44355530-44356129-del*	–	likely pathogenic
2	1	c.12_16delGAACG	p.Asn5Alafs*50	likely pathogenic
3	1	c.630_644del	p.Thr211_Met215del	VUS
4	1	c.956_958del	p.Leu319del	likely pathogenic

cDNA, complementary DNA; ACMG Classification, American College of Medical Genetic Classification.

*confirmed by the сhromosome microarray analysis (CMA), a microdeletion of a region of chromosome 20 from position 44354712 to position 44357770, 3058 bp in size, including exon 1 of the HNF4A gene, was detected.

### 3.6 Treatment of patients with *HNF4A*-MODY

**Figure 2:**
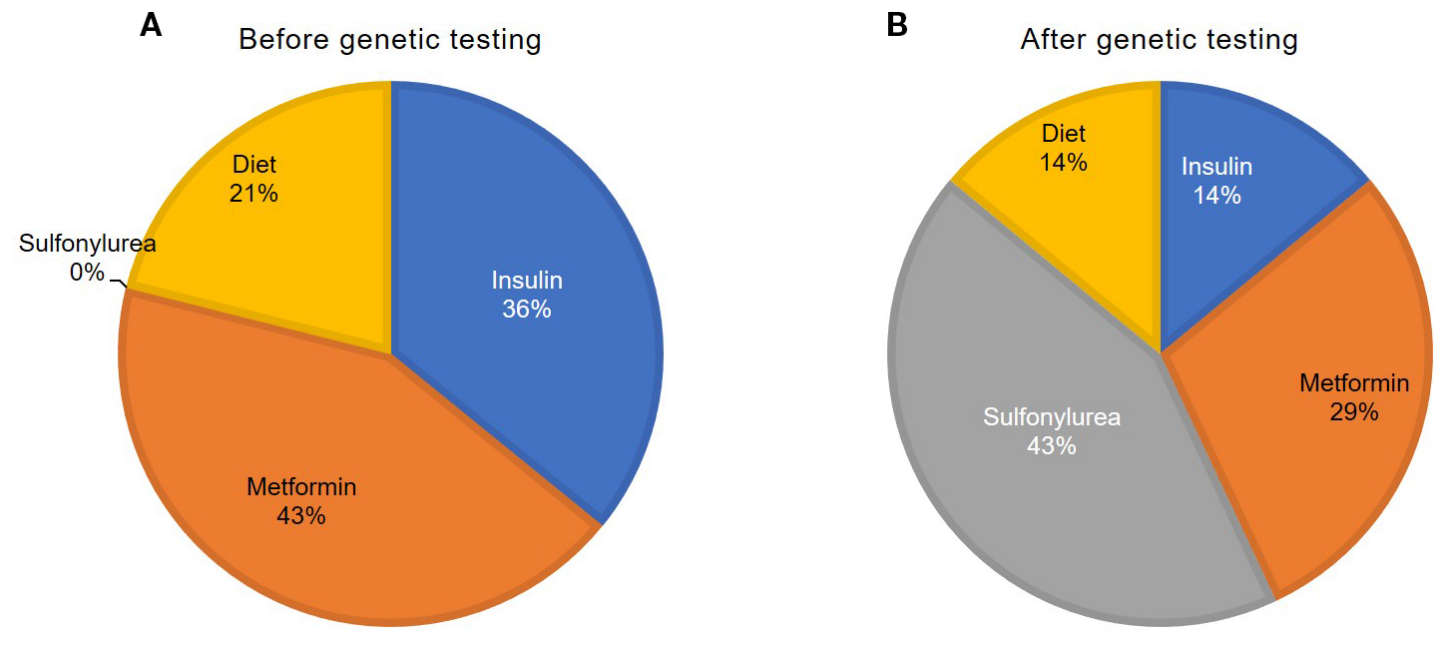
Treatment of the patients with *HNF4A*-MODY before **(A)** and after **(B)** genetic testing.

In a newly diagnosed DM, 21% (*n* = 3) of the patients began an insulin therapy (0.3 U/kg/day [0.3; 0.4]), 43% (*n* = 6)—a metformin therapy (1000 mg/day [1000; 1625]), and 36% (*n* = 5) were treated only with a low-carbohydrate diet (**Figure 2**).

After verification of the genetic cause of *HNF4A*-MODY, 43% of the patients (*n* = 6) were transferred to a sulfonylurea medication therapy, including 7% (*n* = 1) with a complete withdrawal of insulin. Four patients were administered with glimepiride (1.0 mg/kg/day [1.0; 2.3]), and 2 were administered with gliclazide (45 mg/day). 14% (*n* = 2) continued to be administered with insulin (0.48 U/kg/day). Sulfonylurea therapy was unsuccessful due to glycemia deterioration in a case. 29% of the patients (*n* = 4; these were obese patients) continued to be administered with metformin (1000 mg/day [875; 1375]).

After genetic testing and additional examination, 14% of patients (*n* = 2) did not require medication therapy.1 year after switching patients from insulin to SU preparations, the level of HbA1c was 6.7% [6.3; 7.2]. a year after switching to SU from insulin.

## 4 Discussion

*HNF4A*-MODY is a rare MODY subtype in the pediatric population (11). In Russia, among 807 registered probands diagnosed with MODY under the age of 18, 15 patients were diagnosed with the *HNF4A*-MODY subtype. Thus, the frequency of occurrence of *HNF4A*-MODY in children diagnosed with MODY is 1.8%, which corresponds to the fourth place after *GCK*, *HNF1A-*,and *ABCC8-*MODY.

It is noteworthy that deletions were detected in one-third of our patients, while missense variants predominated in other studies (14). As in other studies, our patients more frequently had nucleotide sequence variants in exon 8 (43%) (15).

The *HNF4A* nucleotide sequence variant c.439G>A:p.Val147Ile was confirmed as benign according to ACMG criteria (the patient was subsequently excluded when calculating the study results) in a case. The variant of p.Val147Ile (rs142204928) has been previously described and identified in a girl with diabetes onset at the age of 17 and a family medical history with DM through an exome-wide association study of 28 patients with an early onset of DM from Seoul (16). The same variant was previously identified in three Filipino brothers and sisters from a family with MODY (17). However, this variant has also been identified in two individuals without DM (18) and detected in **>**1% of the chromosomes of South Asia and 20 ExAC homozygotes (19), the majority (17) found in South Asia. This variant is recognized as having an unknown clinical sense and is categorized as likely benign; further studies are required to fully study out its clinical sense. Another variant, p.Thr211_Met215del, was defined as a variant of an unknown clinical sense (VUS) according to the ACMG criteria. These patients exhibited a characteristic *HNF4A*-MODY phenotype, so we believe that both variants can lead to the development of MODY. The remaining 13 variants of the nucleotide sequence were pathogenic or likely pathogenic.

Most of our patients did not have typical clinical signs of DM at the time of *HNF4A*-MODY onset, and the levels of HbA1c were not significantly elevated. Endogenous basal insulin secretion was preserved, but glucose-stimulated insulin secretion was reduced one and a half years after the diagnosis of DM, which is consistent with data from the studies conducted in adult patients (20, 21).

As in other studies (20, 22), our patients had a high intrafamilial concentration of DM (57.1%); *HNF4A*-MODY was confirmed in 35.7% of the probands**’** parents.The median age at the time of diagnosis of DM differed significantly between parents and probands, which is consistent with the literature data on an earlier manifestation of clinical signs of *HNF4A*-MODY (14) in offspring. Patients with *HNF4A*-MODY may have different insulin secretion disorders throughout life (23); as an example, in the neonatal period, some patients exhibit hyperinsulinemic hypoglycemia (20), and later, in the period of adolescence, MODY diabetes develops as a result of depletion of the β-cell function (21, 23). In our case, such a disease course was observed in only one patient (7%). Probably, the absence of antenatal hyperinsulinism, macrosomia, and hypoglycemia in the neonatal period indicates a more pronounced defect of the β cells, which may possibly explain an earlier onset of DM in our group of patients (20, 21, 23, 24).

It was shown that in patients with congenital macrosomia and a defect in the *HNF4A* gene, DM developed less frequently and later. In this case, DM developed at a later age (an increase in birth weight by 1 kg reduced the risk of DM development by 30%), which demonstrates a correlation between fetal and adult insulin secretion (25).

Chronic complications of DM were not diagnosed in our patients, which is explained by a short duration of DM. A low-carb diet often sufficiently controls hyperglycemia at early stages of *HNF4A*-MODY (20). This diet was effective at hyperglycemia diagnosis in one-third of our patients. However, half of these patients required treatment in 1.2 years after the genetic testing was performed. The first-line therapy for *HNF4A*-MODY is SU-derived medications (20), administered in low doses (24). A sulfonylurea therapy was successfully initiated in 43% of our patients. The factors that predict the effectiveness of the sulfonylurea treatment for *HNF4A*-MODY include a shorter duration of DM and a lower level of HbA1c and BMI (26). This confirms the need for an early genetic diagnosis and a timely prescription of the correct treatment.

Kyithar et al. (27) reported a successful treatment of two patients with *HNF4A*-MODY with the help of metformin. Four of our obese patients continued with the administration of metformin after the genetic testing, with a reasonable effectiveness in terms of the glycemic control. However, the standard therapy is not effective in all patients with *HNF4A*-MODY. When other medications are ineffective, as a rule, insulin is prescribed (20).

It is reported in the literature that successful treatment results were obtained with the use of SGLT2 inhibitors in combination with SU (28). The mutual regulatory influence of *HNF4A* and *HNF1A* explains the similar pathogenetic mechanisms of *HNF4A*-MODY and *HNF1A*-MODY (22, 29), which suggests the effectiveness of the same medications in these subtypes of MODY. Thus, recent studies have shown the efficacy of a dual receptor agonist of GIP/GLP-1 (tirzepatide) in patients with *HNF1A*-MODY (30–33). We had no experience of the use of GLP-1 RA or dual GIP/GLP-1 RA or SGLT2 inhibitors in our patients. Accordingly, it will be interesting for future studies in the field of *HNF4A*-MODY therapy to evaluate the efficacy of this group of medications. An interesting direction for future research is the use of GLP-1 RA and dual GIP/GLP-1 RA in children with *HNF4A*-MODY and an evaluation of their effect on improving the endogenous insulin secretion in patients with *HNF4A*-MODY with a long-term application. Moreover, the first positive results have already been obtained (32, 33).

## 5 Conclusion

*HNF4A*-MODY is a rare pediatric DM type. This MODY subtype should be suspected when patients have an incidental DM diagnosis, preserved insulin secretion, absent or low insulin requirements, and a negative pancreas insular autoimmune antibody test. The aggravated family medical history of DM is also typical. Macrosomia is observed in approximately half of neonates with *HNF4A* defects. In early disease stages, children with *HNF4A*-MODY may achieve the target glycemic values through adherence to a low-carb diet. If hypoglycemic therapy needs to be prescribed, sulfonylurea medications may be recommended as a first-line therapy; if they are ineffective, insulin therapy should be initiated.

### 5.1 Сlinical significance of the results

A large group of patients with HNF4A-MODY syndrome in the pediatric population is presented. The study describes the heterogeneity of phenotypic manifestations, molecular genetic features and therapeutic options associated with this disease.

### 5.2 Limitations of the study

The small sample size is a limitation for our study, which is due to the rarity of this pathology.

### 5.3 Directions for further research

Considering the disease median duration in the examined patients of 1.2 years, a further prospective observation of the patients is necessary to assess evolution of the degree of CMD, frequency of the complications of DM, and the treatment effectiveness.

## Data Availability

The original contributions presented in the study are included in the article/supplementary material. Further inquiries can be directed to the corresponding author.
